# Study of the Compression Behaviour of Ti6Al4V Trabecular Structures Produced by Additive Laser Manufacturing

**DOI:** 10.3390/ma12091471

**Published:** 2019-05-07

**Authors:** Matteo Benedetti, Johanna Klarin, Frida Johansson, Vigilio Fontanari, Valerio Luchin, Gianluca Zappini, Alberto Molinari

**Affiliations:** 1 Department of Industrial Engineering, University of Trento, via Sommarive 9, 38123 Trento, Italy; matteo.benedetti@unitn.it (M.B.); johanna.klarin@gmail.com (J.K.); Johanssonfrida@hotmail.com (F.J.); vigilio.fontanari@unitn.it (V.F.);; 2 School of Engineering, University of Jonkoping, 55318 Jönköping, Sweden; 3 Eurocoating SpA, viale Dante 300, 38057 Pergine Valsugana, Trento, Italy; valerioluchin@eurocoating.it (V.L.); gianlucazappini@eurocoating.it (G.Z.)

**Keywords:** trabecular structures, additive manufacturing, mechanical properties, Ti alloy

## Abstract

The aim of this paper was to investigate the compression properties of several trabecular structures produced by additive laser manufacturing of a Ti6Al4V, having different densities and unit cells. Filling space structures were investigated, with different unit cells characterized by both bending-dominated and stretching-dominated behaviour. The stiffness and yield strength were correlated to relative density according to the Gibson and Ashby model. For a constant porosity, the stiffness and the yield strength varied between two extremes represented by the cubic structure (stretching-dominated deformation) and the cross structure (bending-dominated deformation). The properties of the deformed structures did not differ substantially from those of the regular structures. Only in the cubic structure did distortion enhance the contribution of bending to deformation and both stiffness and strength decreased. Cross structures displayed the highest strength at constant stiffness than the others, since they are characterized by the most favourable orientation of the struts.

## 1. Introduction

The additive laser manufacturing (ALM) techniques are finding growing applications in the production of orthopaedic and spinal implant components made of titanium alloys, especially Ti6Al4V. The well-established ability to manufacture parts with high geometrical complexity and mechanical properties, combined with the possibility of creating highly porous structures for preventing the stress-shielding effect and promoting the bone in-growth, represent the major benefits of such techniques [1]. The stress-shielding effect derives from the mismatch between the stiffness of the metallic implant (Young’s modulus of the Ti6Al4V alloy is 110 GPa) and of the bone (15–20 GPa for the cortical bone, 1–5 GPa for the more porous, trabecular bone). Under these conditions, the load is nearly completely carried by the metallic implant; the surrounding bone is then “stress-shielded” and experiences low levels of stress, which can lead to resorption of the bone, impairing the stability of the implant. To prevent such an effect, a large number of pores/voids are introduced into the metallic implant to decrease its stiffness down to the level of the hosting bone. Among the various available technologies reviewed by Ryan et al. [2], selective laser melting (SLM) and electron beam melting (EBM) have the advantage of broad design freedom in manufacturing cellular and trabecular structures mimicking the bone structure, in the form of a monolithic part, of a coating on a full density substrate, or, as recently proposed, of a functionally graded material (FGM) [3,4,5,6]. The porosity introduced to meet the stiffness requirement has an additional positive effect in terms of bone in-growth; the bone tissue precursors (osteoblasts and cell fluids) are able to permeate the voids and to create new bone structures inside the porous parts, thus leading to a better stabilization of the implant component and a reliable long-term fix [7]. The effect of pores on bone in-growth depends on their size and on the architecture of the porous structure. Several studies in the literature report the effect of pore size on different materials, sometimes leading to conflicting results, as recently discussed by Montazerian et al. [8]. In a recent paper, Taniguchi et al. [9] investigated the effect of a pore size of 300, 600, and 900 μm of a titanium implant on bone in-growth in rabbits and concluded that a pore size of 600 μm is the optimum compromise between rapid bone in-growth and mechanical strength.

Mechanical strength is an important issue since porosity not only decreases the stiffness, but also the yield strength of the material. Most of the papers in the literature investigate the compression behaviour of porous structures with different architectures. The correlation between relative density *ρ_r_* and stiffness and strength of the cellular structures depends on the deformation behaviour. According to Ashby [10], relative modulus E/E_0_ and relative strength σ/σ_0_ scale as *ρ_r_* in ideally stretch-dominated structures and as (*ρ_r_*)^2^ and (*ρ_r_*)^1.5^, respectively, in ideally bending-dominated structures. The author concluded that bending-dominated behaviour must be prevented if stiffness and strength at low weight are sought, as a clear indication to designers. Ahmadi et al. [11] reported an extensive study on five different structures with six different densities, confirming that the Young’s modulus and the compression strength may be correlated to density by the Gibson–Ashby model [12]. The coefficients of the power law relation for the different structures are very different and deviate from the theoretical values, so that different mechanical properties may be obtained for the same porosity by varying the unit cell of the porous material. Zadpoor and Hedeyati [13] reviewed the analytical relations proposed in the literature, concluding that more research is needed to develop relations usable for the design of porous structures. Nevertheless, the Gibson–Ashby relation was used by Jette et al. as a basis for the design of a femoral stem incorporating a diamond cubic lattice structure [14] and by Simoneau et al. to design a porous metallic femoral stem [15].

Most of the papers investigate regular structures based on different unit cells, varying density, strut size, and pore size. Recently, structures based on the triply periodic minimal surface were investigated as an alternative solution to achieve the optimal combination between cell permeability and mechanical properties [8].

In the present work, nine different trabecular structures were produced by additive laser manufacturing with different values of density obtained by varying the strut diameter and the pore size. Filling space structures were considered, with different unit cells characterized by both bending-dominated and stretching-dominated behaviour. A certain degree of distortion was introduced in some cases to modify the regularity of the trabecular structure. The aim of the work was to carry out a preliminary investigation of the mechanical properties of a large variety of structures in order to identify any possible candidate to achieve the optimum combination of stiffness and strength under compression loading. Even though bone in vivo is subject to a complex loading state (combining compression, tension, and shear), compression tests were carried out for such a preliminary study. The Gibson–Ashby model was used to correlate the mechanical properties to relative density.

## 2. Materials and Methods

Cylindrical specimens of 30 mm height and 15 mm diameter (the geometry is compliant with standard ISO 13314:2011 [16]) were fabricated starting from a spherical Ti6Al4V powder with a particle size in of 15–45 μm, using a Renishaw AM250 SLM machine equipped with a pulsed laser with a nominal power of 200 W.

The scan speed and the layer thickness were kept constant in the production of all the specimens. A stress relief treatment was carried out at Eurocoating under proprietary conditions to relieve residual stresses and transform the martensitic as-built microstructure.

**Figure 1:**
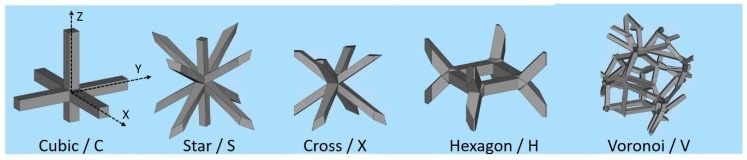
The five base architectures investigated.

The trabecular structures were produced with the five different architectures shown in Figure 1; the building direction in ALM (*Z*-axis) is parallel to the vertical direction in the figure.

The cubic (C), star (S) and cross (X) structures were also produced by introducing a slight distortion on the angles between struts; they are called “irregular” in the following (Ci, Si, Xi). The nodes in these structures lie in a spherical volume with a defined radius centred on the position of the nodes in the regular structures. A third variant of the cubic structure was finally obtained by introducing a random distortion of the angles (to such an extent that a unit cell cannot be recognized) and a random arrangement in the space through a fully random shift of the nodes; this is called “random” in the following (Cr).

**Figure 2:**
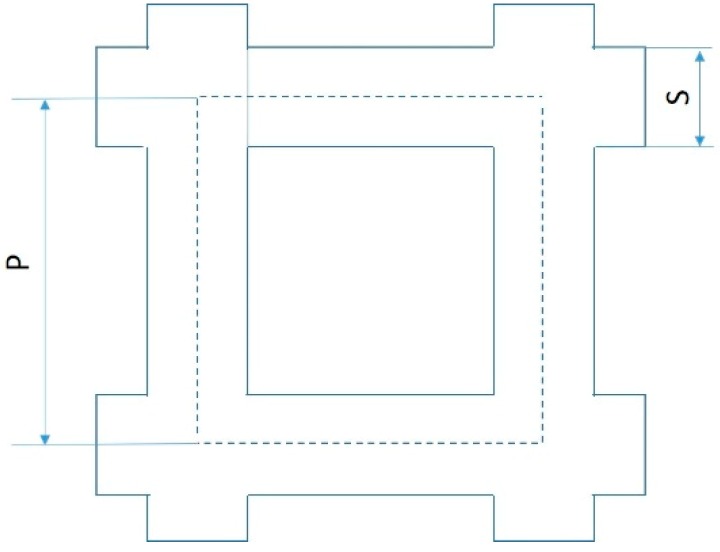
Schematic representation of the cubic (C) structure: P is the pore size, S is the strut diameter.

Three batches were produced by combining different average pore size, P, and strut diameter, S, as follows (as per nominal Computer-Aided Design—CAD data) with reference to Figure 2, relevant to the C structure:

- Batch 1, 700 μm pore size and 200 μm strut diameter;
- Batch 2, 1500 μm pore size and 500 μm strut diameter;
- Batch 3, 1500 μm pore size and 200 μm strut diameter.

The pore size is the diameter of the voids inside the unit cell in the undeformed structures; for instance, in structure C it corresponds to the minimum distance between the axis of two parallel struts. The resulting nominal porosity, determined as the ratio between the volume of the CAD model and the volume of the full element, is reported in Table 1 for each structure.

The density, *ρ*, was determined from the mass (measured with a precision balance, 0.0001 g) and the volume (measuring dimensions with a micrometric calibre, 0.001 mm) on seven specimens for each structure. Before weighing, the specimens were cleaned for 5 min in ultrasounds in ethanol, followed by drying in an oven for 2 h at 120 °C. Assuming a theoretical density of *ρ*_0_ = 4.42 g/cm^3^, porosity was determined as

(1) $$\mathrm{porosity}=100\left(1-\frac{\rho }{\rho_{0}}\right).$$

**Figure 3:**
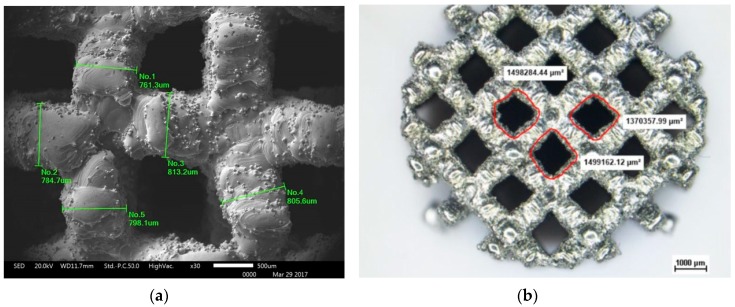
Examples of the determination of the strut and the pore size in the C structure. (**a**) strut size, (**b**) pore size.

The strut size was verified at the scanning electron microscope (SEM) by measuring the diameter of twenty struts for each structure. The pore size was also verified by measuring the area and the minimum Feret diameter, D_min_, at the stereographic microscope. The minimum Feret diameter is indicative of the minimum dimension of the pores available for the migration of osteoblasts and formation of new tissues inside the construct. For a more precise definition of the pore size, the elaboration of 3D data obtained by a tomography should be carried out, but this analysis is out of the scope of the present work. Examples of the two measures are shown in Figure 3.

The microstructure of the heat-treated specimens was investigated with the light optical microscope after metallographic preparation and etching with Kroll’s reagent.

Monotonic compression tests were carried out at room temperature (20 ± 3 °C) according to ISO 13314:2011 [16] using a servo-hydraulic testing machine operated under stroke control with a constant cross head speed of 1 mm/min. The specimen deformation was determined from the displacement between the compression plates (precision 0.005 mm). The stiffness of the testing machine was measured, resulting 143 kN/mm. The tests were stopped either when the structures collapsed due to the fracture of struts, or when 50% deformation was reached, or when the maximum force available (90 kN) was reached. In addition, cyclic compression tests were performed by applying a triangular wave shape with the same testing machine under load-control.

The ISO standard dictates the procedure to determine the following parameters representative of the compression behaviour:

- The quasi elastic gradient E_mon_, i.e., the slope of the linear step in the compression curve;
- The yield strength σ_y0.2_;
- The first maximum compressive strength σ_max_;
- The plateau stress σ_pl_, as the mean value of the stress in the 20–30% deformation range;
- The cyclic Young’s modulus E after its stabilization during loading/unloading cyclic tests in a stress range between 20% and 70% of σ_pl_.

Since not all the curves displayed the plateau stress, an alternative method was adopted to determine the Young’s modulus after its stabilization. This procedure will be described in the next section.

## 3. Results and Discussion

### 3.1. Porosity and Microstructure

**Figure 4:**
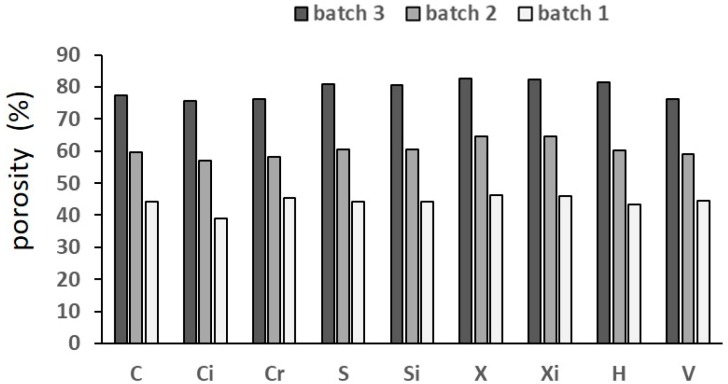
Porosity of the structures investigated, as calculated from Equation (1); scatter ±1%.

Figure 4 shows porosity of the specimens; the average value of five measures are reported, the scatter being ±1%.

**Figure 5:**
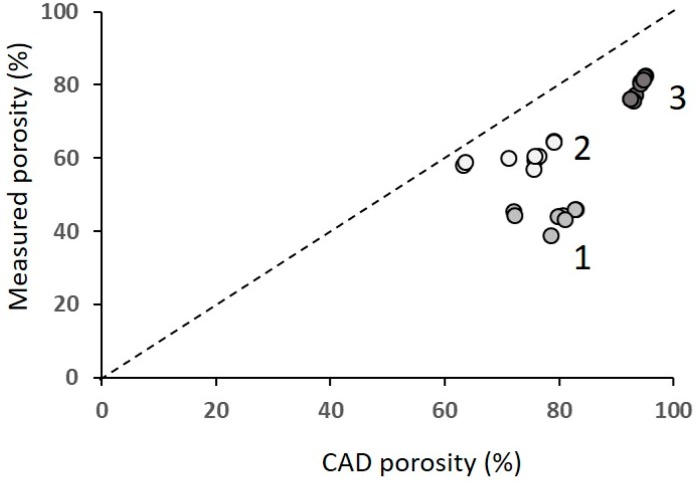
Actual porosity versus nominal (CAD) porosity. Numbers refer to batches: 1—highest density; 2—intermediate density; 3—lowest density.

Porosity increased from batch 1 to 2 to 3 and was slightly affected by the structure. The cross (X) and hexagon (H) were the most porous structures and the cubic (C) and the voronoi (V) were the least porous structures. The measured porosity was much lower than the designed porosity (Table 1); this comparison is shown in Figure 5.

The difference was much larger in batch 1 (actual porosity is from 50% to 63% of the designed porosity) than in the other batches (from 80% to 93%, with no systematic difference between 2 and 3). Table 2 reports the mean values and the standard deviations of the measurements of the strut diameters and the pore size that were performed both on the base and the lateral surface, corresponding to the XY and the XZ plane, respectively. Data relevant to the nine structures were averaged, since the different architectures did not affect the measured size of the pores and of the struts.

The actual strut thickness was larger than the as-designed CAD value, in particular on the lateral surface (XZ plane). On this plane, in fact, the data referred to vertical and inclined struts, while those on the base surface (XY plane) were all horizontal. The oversizing tendency displayed by the struts parallel to the building direction was already reported by Kasperovich et al. [17] and imputed to strut over-melting. On average, the strut diameter was about 250 µm larger on the XY plane and about 300 µm on the XZ plane. This discrepancy was mainly due to the laser beam size and to the presence of unmelted particles bonded to the struts. As a consequence, the pore size was smaller than designed and porosity was, in turn, smaller. There is no evident correlation between the actual measures of the pore and the value from the CAD data, since, as explained in the previous section, the minimum Feret diameter does not exactly correspond to the information delivered by the design software.

**Figure 6:**
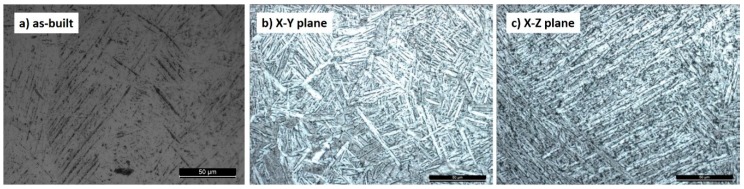
The microstructure of the Ti6Al4V alloy: (**a**) As-built and (**b**,**c**) after heat treatment.

Finally, Figure 6 shows an example of the microstructure in the as-built condition (Figure 6a) and after heat treatment (Figure 6b,c). The as-built microstructure was martensitic and was transformed into stable alpha/beta after heat treatment [18]. This was the same in all the specimens produced, without any significant microporosity in the struts (less than 1% as determined by image analysis on unetched metallographic sections).

### 3.2. Compression Tests

**Figure 7:**
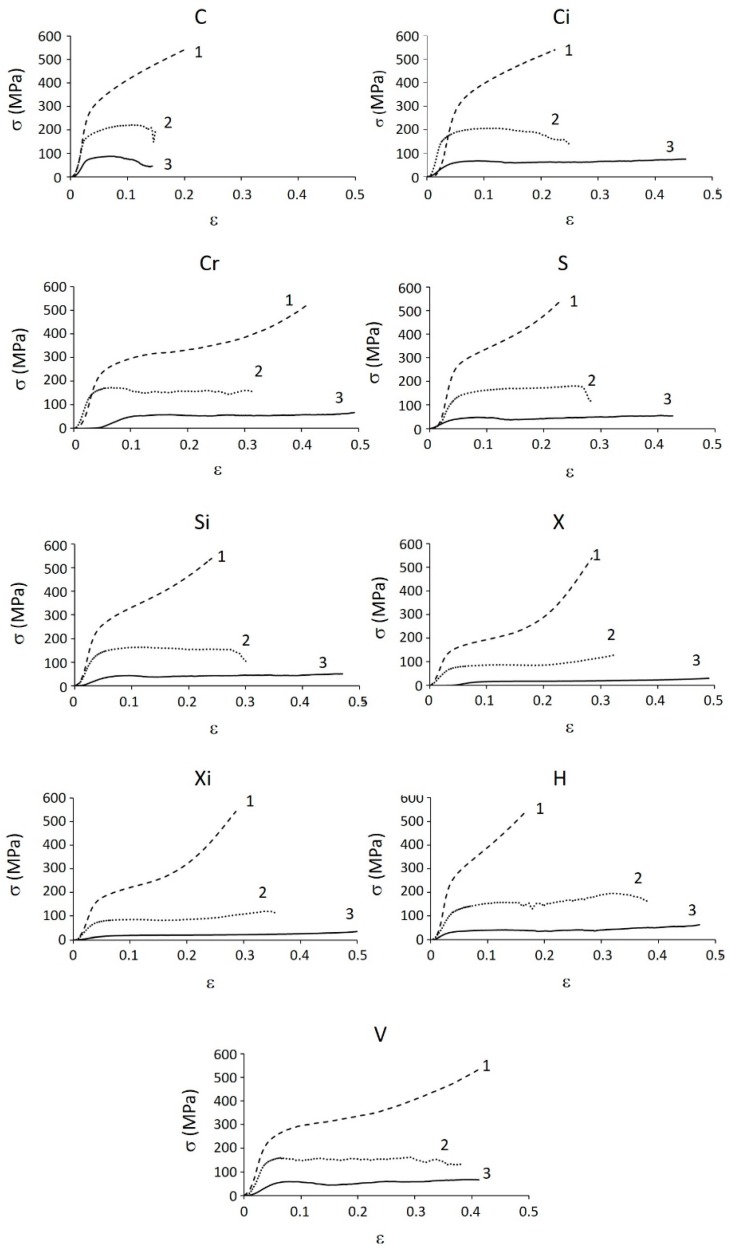
Examples of the compression nominal stress–nominal strain curves. Numbers refer to batches: 1—highest density; 2—intermediate density; 3—lowest density.

Figure 7 shows one example for each structure of the compression nominal stress–nominal strain curves; the porosity increases from 1 to 3 and strength decreases accordingly.

In comparison to the stress–strain curves of foams referred by the ISO 13314:2011 standard [16], structures of batch 1 displayed neither a first maximum compressive strength nor a plateau, since a monotonic densification occurs above the yield point. This result was due to the “low” porosity that made the compression behaviour very different from that of the foams. Hakamada et al. [19] justified this behaviour with the overlapping of the plateau and the densification regions. Nonetheless, a more convincing interpretation was obtained by invoking the low slenderness ratio of the trabeculae, which allowed the plastic deformation to prevail over the mechanical collapse due to instability. In all these cases, the curves reached the maximum available force (90 kN), corresponding to about 510 MPa.

On the contrary, due to the very high porosity, all the structures of batch 3 displayed the typical deformation behaviour of cellular materials with a more or less extended plateau region up to the onset of densification, with the exception of cubic C (the test was interrupted after the collapse of some vertical struts). The onset of a first maximum compressive stress peak indicated that strain softening occurred at the beginning of the plateau [20]. The curves of structures of batch 2 were very similar to those of batch 3, even if four of them displayed the premature collapse of vertical struts (C, Ci, S, Si). In some cases, the curves displayed the initial toe (well evident in Cr-3 and in X-3, for example); the corresponding deformation was subtracted from the total one to elaborate the curves for the determination of the significant parameters described in the previous section.

### 3.3. Mechanical Properties

**Figure 8:**
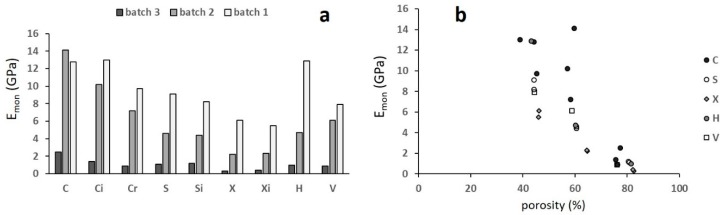
(**a**) The quasi elastic gradient and (**b**) its correlation with porosity; the letters refer to different structures. Scatter ±1 GPa.

Figure 8 shows the quasi-elastic gradient E_mon_ (Figure 8a) and its correlation to porosity (Figure 8b).

**Figure 9:**
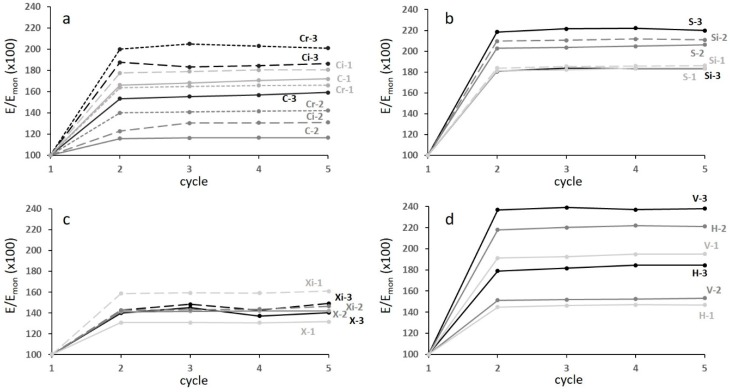
The evolution of the normalized Young’s modulus during cyclic tests. The letters refer to structures and numbers to batches. (**a**) Cubic, (**b**) star, (**c**) cross, (**d**) hexagon and voronoy.

The quasi-elastic gradient decreased with increasing porosity and the correlation with porosity (Figure 8b) was highly scattered. This was due to the poor stability of the trabecular structures during the first loading cycle, in particular for the highly porous ones. For this reason, the standard suggests the stabilization of the mechanical behaviour by carrying out loading–unloading cycles, using the plateau stress as a reference to determine the stress interval for the cyclic tests. Since not all the curves shown in Figure 6 displayed such a stress, an alternative approach was used to determine this interval. This approach was formed from the observation that structures of batches 3 and 2 showed the plateau starting at a deformation of about 15%. The stress corresponding to this deformation was determined and five cyclic tests were carried out between 30% and 70% of this stress in all specimens. The results of the cyclic tests are shown in Figure 9. In each diagram, the evolution of the slope E of the stress–strain curves is reported with reference to the quasi-elastic slope E_mon_ that was normalized at 100.

**Figure 10:**
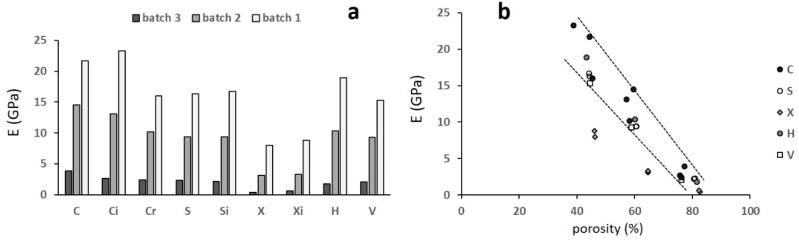
(**a**) The Young’s modulus and (**b**) its correlation with porosity. Scatter ±1 GPa. The letters refer to structures and the dashed lines in (**b**) indicate the width of the scatter.

In all the structures, the slope of the stress–strain curves increased from the first to the second cycle and reached a plateau, indicating that the mechanical stabilization was achieved already after the first cycle. The increase in stiffness can be interpreted as follows: During the first loading cycle, the stress/strain field was inhomogenously distributed in the structure, the largest part of it being loaded in the elastic regime, while localized plastic deformation occurred at the junction between the struts. During the subsequent loading cycle, the elastic regime was extended by strain hardening, resulting in an apparent increase in the structure’s stiffness. Moreover, a further contribution to this increment may be given by the closing effect exerted by the first loading cycle on the internal porosity of the trabeculae. Even in this case, there was no correlation between the increase in stiffness and the porosity, still due to the high scatter of the quasi-elastic gradient. The Young’s modulus E was better correlated to porosity, as shown by Figure 10b.

Still with some scatter, the experimental points depict a band (Figure 10b), with the only exception of structures X that are more compliant with same porosity, due to the lack of struts parallel to the loading direction whereby the deformation behaviour changes from stretching- to bending-dominated. The regular and irregular structure C lie in the upper part of the band due to their particular architecture that maximizes the contribution of stretching to deformation [8].

The yield strength σ_y0.2_ and the first maximum compressive strength σ_max_ are the two main properties representative of the strength of foam materials. While the former can be easily determined from the stress–strain curves, the latter is not present in all the structures. Looking at the curves relevant to structures of batches 2 and 3 where a sharp peak stress is visible, it can be noted that σ_max_ occurs at about 0.04 offset. This value will be assumed to define σ_max_ for all the structures.

**Figure 11:**
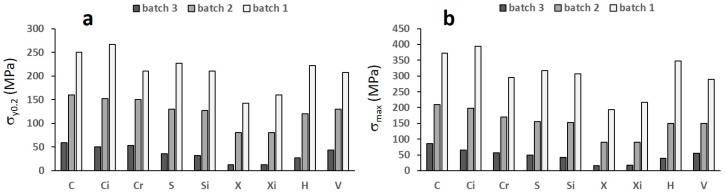
(**a**) The yield strength and (**b**) the maximum compressive strength. Mean scatter is 10 MPa and 12 MPa, respectively.

**Figure 12:**
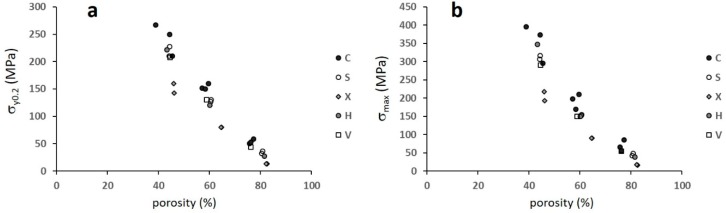
(**a**) The yield strength and (**b**) the maximum compressive strength versus porosity. The capital letters refer to structures.

Figure 11 reports σ_y0.2_ and σ_max_, while Figure 12 shows their correlation with porosity.

Comparing the structures within the single batches, i.e., with similar ranges of porosity (Figure 10), it can be noted that structures C and Ci possess the highest, while structures X and Xi show the lowest strength. All data depict one single correlation with porosity, with the exception of the least resistant structures X and Xi (Figure 12).

**Figure 13:**
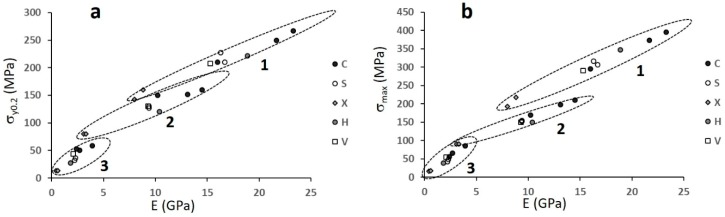
(**a**) Yield strength and (**b**) maximum compressive strength versus Young’s modulus for all the structures investigated. The capital letters refer to structures, numbers, and circled groups to batches.

Figure 13 plots σ_y0.2_ and σ_max_ against the Young’s modulus. The aim of such a representation is to verify the existence of any optimal structure–porosity combination resulting in a maximum strength at any given stiffness.

The three batches depict three different relationships between strength and stiffness, with some overlapping in two Young’s modulus intervals: 3–5 GPa and 8–15 GPa. In both cases, the X structures, albeit being the weakest among the ones with similar porosity (Figure 12), exhibit the highest strength among the structures with similar stiffness.

**Figure 14:**
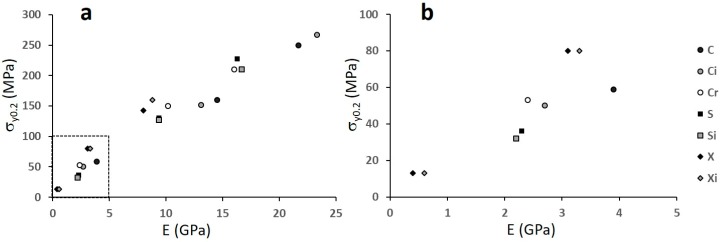
Yield strength versus Youngs modulus; the graph (**b**) is an enlarged inset of the dashed area in the graph (**a**). The letters refer to structures.

The effect of introducing some distortion into the structure is visible in Figure 14, where only data relevant to structures C, S, and X are shown, differentiating the regular structures from the distorted ones (irregular) and, in the case of structure C, also from the random one.

As it might be expected, the modification of the cell architecture from the regular one exerts a significant effect on the cubic structure only, since distortion and randomization cause a strong deviation from the stretching-dominated behaviour typical of these structures. The distortion of the cubic cell lowers the mechanical performances and only at low level of porosity (i.e., at the highest levels of strength and stiffness) do the two structures C and Ci seem to be similar.

### 3.4. The Model for Stiffness and Strength

As mentioned in the introduction, the Gibson–Ashby equation is often used to correlate stiffness (Equation (2)) and yield strength (Equation (3)) to density in highly porous structures.

(2) $$\frac{E}{E_{0}}=C_{1}{\left(\frac{\rho }{\rho_{0}}\right)}^{n_{1}},$$

(3) $$\frac{\sigma_{y}}{\sigma_{y0}}=C_{2}{\left(\frac{\rho }{\rho_{0}}\right)}^{n_{2}},$$

where *E*_0_ and *_y_*_0_ are relevant to the pore free material, viz. 110 GPa and 831 MPa, respectively, as determined from tensile tests on full density test pieces produced with the same parameters of the trabecular structures. *C*_1_ and *C*_2_ are constants depending on the material and on the cell architecture, and *n*_1_ and *n*_2_ are two other constants depending on the configuration of struts relative to the loading direction. In particular, for foams where bending dominates the deformation behaviour, *C*_1_ = 1 and *n*_1_ = 2, while for foams where deformation occurs by stretching, *C*_1_ = 0.3 and *n*_1_ = 1 [21,22]. Kadkhodapour et al. [23] reported 0.904 and 1.658 for the exponent of the elastic Young’s modulus for cubic and diamond structures that mainly deform by stretching and bending, respectively. Yan et al. [24] investigated the elastic modulus of Gyroid and Diamond Triply Periodic Minimal Surface lattice structures with various densities and report *C*_1_ = 0.19 and 0.17, respectively, and *n*_1_ = 1.71 and 1.64, respectively. They also considered yield strength, reporting *C*_2_ = 1.31 and 1.39, respectively, and *n*_2_ = 1.83 and 1.95, respectively.

**Figure 15:**
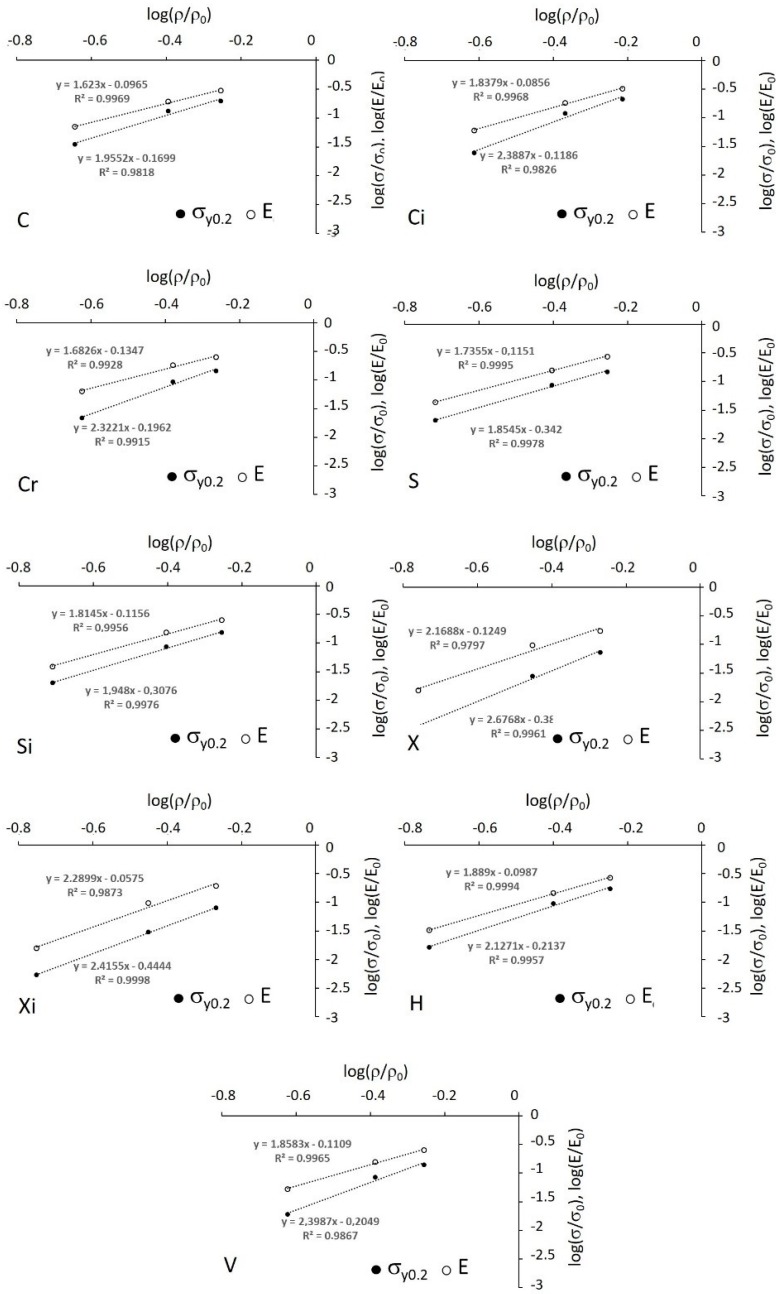
Log–log plot of the Gibson–Ashby model for Young’s modulus and yield strength. The letters refer to structures.

Figure 15 shows the Gibson–Ashby plots in log–log diagrams for the Young’s modulus and the yield strength.

The Gibson–Ashby models apply well to the correlations between stiffness and yield strength and density (porosity), as demonstrated by the correlation coefficients that are reported in Table 3 along with the parameters of Equations (1) and (2).

The parameters of the two equations deviate from those reported in the literature. For instance, structure C and, to some extent, structure Ci are expected to display a stretching-dominated behaviour, but their exponent in the stiffness model is much higher than 1, close to that expected for bending-dominated structures. This unexpected result may be imputed to inevitable misalignment (due to manufacturing and testing issues) between longitudinal struts and loading axis. Accordingly, it was demonstrated in [25] for honeycomb structures that even a misalignment of 5° induces a reduction by 80% in the axial stiffness.

**Figure 16:**
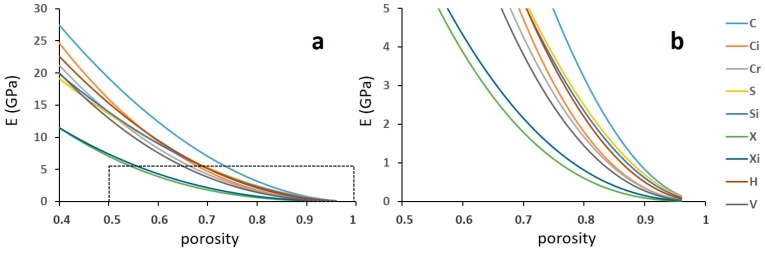
Effect of porosity on the Young’s modulus according to the Gibson–Ashby model. The letters refer to structures. The graph (**b**) is an enlarged inset of the dashed area in the graph (**a**).

**Figure 17:**
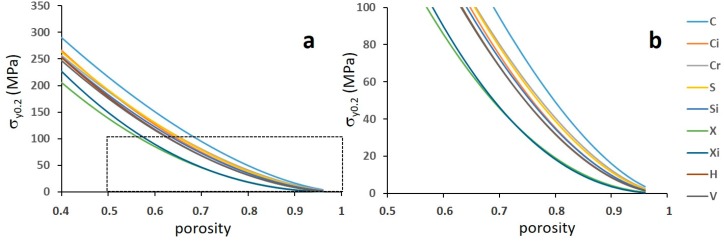
Effect of porosity on the yield strength according to the Gibson–Ashby model. The letters refer to structures. The graph (**b**) is an enlarged inset of the dashed area in the graph (**a**).

In all the remaining structures, the mechanical behaviour is expected to be dominated by bending and, in fact, the corresponding exponent scatters around 2 within quite a large band. The uncertainty in the fitting parameter indicates that a more robust determination thereof would require a larger number of experiments, replicating several tests on different densities. Nevertheless, for a first approximated scrutiny of the phenomenon attempted in the present paper, these parameters are used to draw the diagrams reported in Figure 16 and Figure 17. It can be noted that the Young’s modulus and the yield strength are estimated within a large porosity range able to bring the stiffness to values of practical interest for preventing the stress-shielding effect.

Both stiffness and strength range in large intervals, with the C structure and the two X structures at the upper and the lower boundaries, respectively. All the other structures lie between these with rather small differences.

## 4. Conclusions

The compression properties of several trabecular structures with different density and unit cell produced by additive laser manufacturing of a Ti6Al4V powder were investigated. The main results of the study may be summarized as follows.

Depending on density and cell architecture, stiffness and strength under compression loading vary at large intervals. The slopes of the stress–strain curves increase from the first to the second cycle and stabilize due to the occurrence of localized plastic deformation at the junctions between the struts that extends the elastic regime by strain hardening. The properties are correlated to relative density according to the Gibson and Ashby model, the parameters of the power law equations being dependent on the structure. Using this model, the mechanical properties required by the specific application can be designed defining the structure, the strut, and the pore size that determine density. However, ALM tends to significantly oversize the struts and this has to be taken into account when designing the structures.

From the Gibson–Ashby models, it may be concluded that the cubic structures deviate from the expected stretching-dominated behaviour due to the effect of some misalignment between the vertical structs and the loading direction, which introduces a significant contribution of bending in deformation.

For a constant porosity, the stiffness and the yield strength of the various structures investigated vary between two extremes represented by the cubic structure (C) (smaller contribution of bending) and the cross structure (X) (larger contribution of bending); all the other structures do not display any practical difference, particularly in stiffness.

The properties of the deformed structures do not differ substantially from those of the regular structures, since deformation does not affect the deformation behaviour significantly. Only in the case of the cubic structure, distortion and randomization enhance the contribution of bending to deformation and both stiffness and strength decrease. The properties of the deformed structures are very similar to each other, independent of the cell architecture. The distortion and randomization of the cubic structure is expected to decrease its anisotropy resulting from the orientation of struts that are either parallel or perpendicular to the building direction. This effect will be investigated more deeply in future work.

The X structures display the highest strength at constant stiffness than the others. With reference to the manufacturing process, this structure is characterized by the most favourable orientation of the struts, since they are self-supporting and do not require the introduction of additional supports.

Some of the compression curves (structures C and S and Si) display a sharp decrease of the stress during the plastic deformation, due to the collapse of the vertical struts. This behaviour is highly risky in case of overloading, since it involves the highest probability of the production of fragments that are not accepted in orthopaedic applications. From this viewpoint, all the other structures are preferable.

The present study highlights the behaviour of the investigated structures under a compression loading. The cross structures display the best combination of compression properties. The results will be confirmed by further investigating some selected structures and increasing the variables relevant to the strut size, the pore size, and the resulting density. Moreover, other loading conditions will be explored by carrying out tensile and fatigue tests, as well as tests on the permeability to verify the functionality of the porous structure.

## Figures and Tables

**Table 1:** Nominal percent porosity (as per Computer-Aided Design—CAD) of the structures investigated.

Structure	Batch 1	Batch 2	Batch 3
C	75.6	80.0	93.2
Ci	75.4	78.5	92.9
Cr	63.1	72.0	92.4
S	76.4	80.6	94.2
Si	75.6	79.7	94.2
X	79	82.8	95.0
Xi	79	82.6	94.9
H	71	80.9	94.6
V	63.5	72.2	92.4

**Table 2:** Strut and pore size: Comparison between CAD and experimental values. Standard deviation in parentheses.

Batch	Strut Diameter (μm)			Pore D_min_ (μm)		
	CAD	XY Plane	XZ Plane	CAD	XY Plane	XZ Plane
1	200	420 (50)	510 (30)	700	680 (140)	490 (100)
2	500	770 (40)	830 (60)	1500	1310 (190)	1350 (150)
3	200	440 (40)	500 (20)	1500	1370 (160)	1230 (160)

**Table 3:** Parameters of the Gibson–Ashby models for Young’s modulus and yield strength of different structures investigated.

Structure	Young’s Modulus			Yield Strength		
	*C*_1_	*n*_1_	*R* ^2^	*C*_2_	*n*_2_	*R* ^2^
C	0.68	1.96	0.9818	0.80	1.62	0.9969
Ci	0.76	2.39	0.9826	0.82	1.84	0.9968
Cr	0.63	2.32	0.9915	0.73	1.68	0.9928
S	0.45	1.86	0.9978	0.77	1.74	0.9995
Si	0.49	1.95	0.9976	0.77	1.81	0.9956
X	0.41	2.68	0.9961	0.75	2.17	0.9797
Xi	0.36	2.42	0.9998	0.88	2.29	0.9873
H	0.61	2.13	0.9957	0.80	1.89	0.9994
V	0.62	2.40	0.9867	0.77	1.86	0.9965
